# Adverse Events of PD-1, PD-L1, CTLA-4, and LAG-3 Immune Checkpoint Inhibitors: An Analysis of the FDA Adverse Events Database

**DOI:** 10.3390/antib13030059

**Published:** 2024-07-17

**Authors:** Connor Frey, Mahyar Etminan

**Affiliations:** 1 Department of Medicine, University of British Columbia, 317-2194 Health Sciences Mall, Vancouver, BC V6T 1Z3, Canada; 2 Department of Ophthalmology and Visual Sciences, University of British Columbia, 2550 Willow Street, Vancouver, BC V5Z 3N9, Canada; etminanm@mail.ubc.ca

**Keywords:** immune checkpoint inhibitors, immunotherapy, immuno-oncology, PD-1, PD-L1, CTLA-4, LAG-3, pharmacovigilance, AERSMine, patient safety

## Abstract

This study aimed to identify the 25 most prevalent adverse events (AEs) associated with FDA-approved immune checkpoint inhibitors (ICIs)—specifically, PD-1, PD-L1, CTLA-4, and LAG-3 inhibitors—using data from the FDA Adverse Events Reporting System (FAERS), a publicly available repository of reported drug adverse events, and AERSMine, an open-access pharmacovigilance tool, to investigate these adverse events. For PD-1 inhibitors, the most common AEs were diarrhea, fatigue, and pyrexia, with notable instances of neutropenia and hypothyroidism, particularly with toripalimab and dostarlimab. PD-L1 inhibitors also frequently caused pyrexia, diarrhea, and fatigue, with interstitial lung disease and hypothyroidism showing a class effect, and drug-specific AEs such as hepatotoxicity and chills. CTLA-4 inhibitors predominantly resulted in diarrhea and colitis, with ipilimumab frequently causing pyrexia and rash, while tremelimumab exhibited unique AEs such as biliary tract infection. The LAG-3 inhibitor relatlimab reported fewer AEs, including pyrexia and pneumonia. Rare but significant AEs across all inhibitors included myocarditis and myasthenia gravis. This study provides a detailed overview of the 25 most common AEs associated with ICIs, offering valuable insights for clinical decision-making and AE management. Further research is necessary to elucidate the mechanisms underlying these AEs and to develop targeted interventions to enhance the safety and efficacy of ICI therapy in patients with cancer.

## 1. Introduction

The advent of immuno-oncology has changed the landscape of treatment options available to patients with cancer [1,2]. Of these therapies, the monoclonal antibody-based immune checkpoint inhibitors (ICI) have been the most widely utilized to date for a wide range of malignancies, most notably melanoma, lung, and colorectal cancer, as well as tumours with mismatch repair deficiencies (dMMR) or a high tumour mutational burden (TMB) [3,4,5]. These drugs have been developed against multiple targets including CTLA-4 (cytotoxic T-lymphocyte-associated protein 4), PD-1 (programmed cell death protein 1), PD-L1 (programmed death-ligand 1), and LAG-3 (lymphocyte activation gene 3), key regulators of the immune checkpoint [6,7]. This checkpoint is a complex regulatory system that maintains the balance between activation and inhibition of T-cells to prevent excessive immune responses and autoimmune reactions [8]. CTLA-4 functions during the initial phase of T-cell activation, competing with the co-stimulatory molecule CD28 for binding to CD80/86 on antigen-presenting cells, thereby downregulating T-cell activation [9]. PD-1, expressed on T-cells, B-cells, and myeloid cells, interacts with its ligands PD-L1/2 to dampen T-cell responses, serving as a mechanism of self-tolerance [10]. Similarly, LAG-3 negatively regulates T-cell activation upon interaction with its ligand, MHC II [11]. ICIs target these inhibitory pathways, allowing for an anti-tumour response through a blockade of the interaction between these checkpoints and their ligands.

While the widespread activation of T-cells within the tumour microenvironment can yield remarkable responses in certain patients, it is anticipated that an escalation in immunological activity may precipitate AEs. Existing literature indicates that ICIs cause a broad spectrum of AEs, however, no study to date has reported the most prevalent among all the ICIs [12,13,14,15,16]. The objective of this study is to ascertain the most frequently reported AEs associated with the administration of ICIs in the FDA Adverse Events Reporting System. This work is imperative given the rising utilization of ICIs, coupled with the numerous ongoing trials evaluating novel agents. It’s important to highlight to oncologists, as well as other physicians involved in caring for a patient with cancer, the anticipated AEs of these therapies and to provide appropriate counselling. To the best of our knowledge, this paper is the first to report the commonest AEs corresponding to all the FDA-approved ICIs.

## 2. Methods

The FDA Adverse Event Reporting System (FAERS) serves as a comprehensive repository for documented adverse drug reactions attributed to various pharmaceutical products. These reports are sourced from submissions made by pharmaceutical companies, healthcare professionals, and consumers within the USA. Moreover, FAERS encompasses post-marketing clinical trial data derived from investigations conducted domestically and internationally. Due to the public nature of the data, an ethics committee was not involved in this study. Disproportionality analyses involve the comparative assessment of a reported incidence of a specific AE with a given drug against the background reported rate of AEs associated with all other pharmaceuticals. Using OpenVigil 2.1 (OpenVigil, Kiel, Germany) [17], individual queries were performed to retrieve AEs for the PD-1 drugs pembrolizumab, nivolumab, tislelizumab, cemiplimab, dostarlimab, toripalimab, the PD-L1 drugs avelumab, durvalumab, atezolizumab, the CTLA-4 drugs ipilimumab and tremelimumab, and the LAG-3 drug relatlimab.

For this study, reports submitted from the first quarter of 2017 to the first quarter of 2024 were utilized for analysis. The 25 most frequently reported AEs were extracted. These data enabled the calculation of RORs, which indicate the likelihood of an AE occurring in the presence of drug exposure compared to the likelihood of the same AEs occurring in the absence of that exposure, relative to all other drugs. OpenVigil calculates RORs from FAERS by constructing a 2 × 2 contingency table that includes the number of reports of the adverse event with the drug, the number of reports without the adverse event with the drug, the number of reports with the adverse event for all other drugs, and the number of reports without the adverse event for all other drugs. Statistical significance for each ROR value was determined if the lower-bound value of the 95% confidence interval exceeded 1.00 and the ROR was greater than 2.00. Only AEs with five or more reports each were included. OpenVigil 2.1 incorporates data cleaning procedures, including the removal of duplicate records, rectification of improperly formatted entries, and consolidation of terms referring to the same drug under a single term.

## 3. Results

### 3.1. PD-1

There was a total of 44,224 AEs reported across the 25 most prominent AEs from each of the six FDA-approved PD-1 therapies: 21,276 for pembrolizumab, 22,415 for nivolumab, 78 for cemiplimab, 138 for dostarlimab, 114 for toripalimab, and 203 for tislelizumab (Table 1). The most frequently reported AEs observed across all PD-1 ICIs were diarrhea, fatigue, and pyrexia. Toripalimab was most often associated with diarrhea (ROR = 3.646), followed by dostarlimab (ROR = 1.841). Fatigue was most reported with dostarlimab (ROR = 4.554) and toripalimab (ROR = 1.613). Pyrexia was notably associated with dostarlimab (ROR = 7.126) and pembrolizumab (ROR = 2.822). Hematological AEs such as thrombocytopenia and neutropenia varied in frequency among ICIs. Toripalimab (ROR = 8.838) and dostarlimab (ROR = 7.080) were prominently associated with thrombocytopenia, while tislelizumab (ROR = 23.585) and dostarlimab (ROR = 13.954) were more prevalent for neutropenia. Immune-related AEs were observed across all ICIs, with hypothyroidism being the most prevalent. Pembrolizumab had the strongest signal for hypothyroidism (ROR = 25.930), followed by nivolumab (ROR = 20.911). Other notable Immune-related AEs included pneumonitis, reported most frequently with toripalimab (ROR = 45.859) and pembrolizumab (ROR = 20.024), and colitis, notably associated with nivolumab (ROR = 29.467). Other serious AEs such as myocarditis and adrenal insufficiency were rare but exhibited high reporting odds ratios when present. Dostarlimab (ROR = 143.905) and nivolumab (ROR = 29.085) were notably associated with myocarditis, while adrenal insufficiency showed a strong signal with nivolumab (ROR = 45.012).

There was a total of 12,020 AEs reported across the 25 most prominent AEs from each of the three FDA-approved PD-L1 therapies: 8898 for atezolizumab, 927 for avelumab, and 2195 for durvalumab (Table 2). Pyrexia was the most frequently reported AE for all three drugs, with atezolizumab having the highest signal (ROR = 3.475), followed by durvalumab (2.500) and avelumab (2.422). Diarrhea, fatigue, and anemia were also commonly reported adverse events across all three drugs. Atezolizumab had the strongest signal for diarrhea (ROR = 1.481), fatigue (ROR = 1.016), and anemia (ROR = 4.549). Notably, specific adverse events such as interstitial lung disease, hypothyroidism, neutropenia, pneumonitis, and adrenal insufficiency exhibited high RORs across all three drugs, indicating a potential class effect. For example, interstitial lung disease had RORs of 17.187, 15.905, and 20.135 for atezolizumab, avelumab, and durvalumab, respectively. Furthermore, some AEs were unique to individual drugs. For instance, hepatotoxicity, proteinuria, and ascites were reported only with atezolizumab, while chills, malaise, and peripheral neuropathy were reported only with avelumab. Durvalumab showed unique AEs including myocarditis and pleural effusion.

### 3.2. CTLA-4

There was a total of 14,382 AEs reported across the 25 most prominent AEs from both FDA-approved CTLA-4 therapies: 13,989 for ipilimumab and 393 for tremelimumab (Table 3). Among the AEs, diarrhea and colitis were the most frequently reported for both drugs. Ipilimumab exhibited a higher signal for diarrhea (ROR = 2.870) and colitis (ROR = 61.885) compared to tremelimumab. Pyrexia, rash, and fatigue were also commonly reported AEs with ipilimumab, while tremelimumab showed a lower incidence or absence of these events. Pyrexia had a higher.

ROR with Ipilimumab (ROR = 3.780) compared to Tremelimumab (ROR = 9.327). Certain adverse events such as hypophysitis, adrenal insufficiency, and hepatic dysfunction demonstrated high RORs for Ipilimumab, indicating their association with this drug rather than the class as tremelimumab showed a lower frequency of these events or were absent. Additionally, unique adverse events were reported with each drug. Tremelimumab showed a higher incidence of biliary tract infection (ROR = 4275.104) and gastrointestinal hemorrhage (ROR = 7.875), while ipilimumab exhibited adverse events such as pneumonitis (ROR = 21.095) and dehydration (ROR = 2.835).

### 3.3. LAG-3

There was a total of 43 AEs reported across the 25 most prominent AEs for the FDA-approved LAG-3 therapies relatlimab. Pyrexia was reported twice with a moderate ROR of 5.986, while pneumonia had five reported cases with an ROR of 18.674. Despite their limited occurrences, other adverse events, such as myositis (ROR = 227.604), myasthenia gravis (ROR = 344.496), and infection (ROR = 75.279), showed strong signals. Colitis, pneumonitis, and myocarditis were rare but notable AEs, with colitis and pneumonitis each having one reported event with high RORs of 183.992 and 18.674, respectively. Myocarditis was reported three times with a remarkably high ROR of 292.250. Various rare adverse events, including adrenal insufficiency, hepatic dysfunction, and third-degree heart block, were also reported. Each had one reported case and strong signals. Multiple instances of symptoms like anxiety, hyponatremia, and confusion were reported, with RORs ranging from 10.301 to 36.026. It’s worth noting that this drug is administered together with nivolumab as a combination product, and thus there may be an influence of the PD-1 drug class in these AEs. For example, the ROR for both myocarditis and pyrexia were strongly elevated in both relatlimab and nivolumab.

## 4. Discussion

The advent of immune checkpoint inhibitors has revolutionized cancer treatment, offering promising outcomes for patients across various malignancies [18,19]. However, their widespread use has brought attention to the diverse spectrum of AEs associated with these agents, though some studies suggest a better clinical outcome, particularly when endocrine, dermatological, and low-grade immune-related AEs occur [20,21,22]. In this analysis, we examined the commonest 25 AEs across all the FDA-approved PD-1, PD-L1, CTLA-4, and LAG-3 ICIs, highlighting the most common events seen in these therapies to bring awareness to oncologists, among other clinicians, when treating patients on these drugs.

Across all ICIs, a constellation of common AEs emerged, reflecting the systemic impact of immune modulation. Consistently reported AEs included pyrexia, fatigue, diarrhea, and immune-related AEs such as hypothyroidism and pneumonitis and are congruent with our findings [23,24,25]. These underscore the broad activation of the immune system by ICIs, leading to immune-related inflammation and dysfunction broadly. Additionally, hematological AEs such as neutropenia, thrombocytopenia, and anemia were prevalent, highlighting the potential for bone marrow suppression or immune-mediated cytopenia [26]. Hepatic dysfunction and gastrointestinal AEs, including colitis and hepatotoxicity, further underscored the susceptibility of the liver and gastrointestinal tract to immune-mediated injury [27,28]. Notably, certain AEs exhibited drug-specific patterns, suggesting distinct mechanisms or target organ susceptibilities. For instance, CTLA-4 inhibitors were associated with a higher incidence of colitis and diarrhea, while PD-1 inhibitors showed a strong trend toward immune-related AEs, again, congruent with the current literature [29,30]. While some AEs were rare, their occurrence underscored the need for vigilant monitoring and prompt intervention. Infrequent but significant AEs included myocarditis, adrenal insufficiency, myositis and myasthenia gravis, which carried significant morbidity risks and have been shown to result in an ICI-induced overlap syndrome [31]. While these agents have been demonstrated to cause adverse events, the mechanisms are not clearly understood aside from those that are immune-mediated [20]. This opens the door for studies to focus on the pathophysiology of individual events with the highest incidence, elucidated by studies such as this one.

The FAERS database, while invaluable for monitoring adverse drug events, has several inherent limitations that must be considered. Firstly, there is no certainty that the reported event was caused by the product. The FDA does not require proof of a causal relationship between a product and an event, and the reports may not always contain enough detail to assess an event properly. Additionally, the FDA does not receive reports for every adverse event or medication error associated with a product. Lastly, several factors, such as the duration a product has been on the market and the publicity about an event, can influence whether an event will be reported.

Overall, our findings emphasize the importance of vigilant monitoring for and management of, AEs in patients receiving ICIs. The collaboration between oncologists, and immunologists, among others is paramount to promptly recognize and manage AEs, optimize treatment efficacy, and ensure patient safety. Additionally, ongoing research efforts are warranted to elucidate the underlying mechanisms of AEs and develop targeted interventions to mitigate their impact, thereby maximizing the therapeutic potential of ICIs in the era of precision oncology.

## Figures and Tables

**Table 1:** **PD-1 Immune Checkpoint Inhibitor Adverse Events.** Reported odds ratio (ROR) for the 25 most common adverse events associated with each anti-PD-1 immune checkpoint inhibitor.

Adverse Event	Pembrolizumab		Nivolumab		Cemiplimab		Dostarlimab		Toripalimab		Tislelizumab	
	Events	ROR	Events	ROR	Events	ROR	Events	ROR	Events	ROR	Events	ROR
Diarrhoea	1726	1.961	1867	1.832	16	0.767	10	1.841	**6**	**3.646**	9	1.776
Fatigue	1596	1.430	1351	1.035	36	1.403	11	1.613	9	**4.554**	-	-
Pyrexia	1284	**2.822**	1539	**2.937**	26	**2.466**	5	1.756	6	**7.126**	4	1.504
Thrombocytopenia	1172	**3.898**	452	**2.572**	**-**	**-**	13	**7.080**	-	**-**	15	**8.838**
Hypertension	1114	**3.750**	-	-	-	**-**	-	**-**	-	**-**	4	**2.348**
Rash	1100	1.784	1313	1.848	36	**2.574**	4	1.041	**3**	**2.532**	11	**4.564**
Decreased Appetite	1024	**3.417**	1048	**3.018**	15	**2.142**	-	**-**	**7**	**12.901**	7	**4.081**
Nausea	1023	0.850	989	0.708	20	0.721	6	0.811	5	**2.240**	-	-
Hypothyroidism	977	**25.930**	919	**20.911**	10	**10.615**	-	**-**	6	**81.360**	12	**53.695**
Interstitial Lung Disease	872	**14.053**	877	**12.200**	14	**9.401**	-	**-**	-	**-**	-	-
Asthenia	755	1.438	644	1.055	21	1.749	-	**-**	**6**	**6.298**	5	1.670
Malaise	753	1.078	740	0.914	11	0.681	-	**-**	-	**-**	-	-
Neutropenia	700	**3.619**	-	-	-	-	4	**3.338**	5	**13.954**	27	**23.585**
Renal Dysfunction	663	**5.262**	-	**-**	11	**3.737**	-	**-**	-	**-**	5	**33.065**
Anaemia	659	**2.606**	563	1.916	-	**-**	5	**3.220**	**4**	**8.442**	11	**7.860**
Acute Kidney Injury	657	**2.94**	625	**2.413**	10	1.927	-	**-**	-	**-**	-	-
Pneumonitis	655	**20.024**	897	**24.363**	19	**24.113**	7	**33.482**	3	**45.859**	-	-
Dyspnoea	651	0.794	811	0.858	20	1.071	8	1.621	**3**	1.935	-	-
Colitis	650	**14.416**	1541	**29.467**	10	**9.242**	-	**-**	-	**-**	-	-
Pneumonia	643	1.517	834	1.710	32	**3.358**	5	1.929	-	**-**	4	1.652
Vomiting	601	0.87	616	0.77	13	0.819	6	1.431	4	**3.111**	-	-
Arthralgia	562	0.984	563	0.852	13	0.992	3	0.854	-	**-**	-	-
Weight Decreased	489	1.267	641	1.443	-	**-**	4	1.695	-	**-**	-	-
Hepatic Dysfunction	483	**9.536**	1406	**10.559**	21	6.895	-	**-**	**8**	**27.732**	-	-
Peripheral Neuropathy	467	**3.569**	-	-	-	-	-	**-**	-	**-**	-	-
Pruritus	-	-	650	1.157	20	1.805	-	**-**	5	**5.606**	-	-
Adrenal Insufficiency	-	-	644	**45.012**	-	-	-	**-**	-	**-**	-	-
Myocarditis	-	-	460	**29.085**	17	**49.943**	4	**43.709**	**4**	**143.905**	-	-
Headache	-	-	425	0.377	13	0.584	-	**-**	-	**-**	-	-
Sepsis	-	-	-	**-**	15	**4.358**	-	**-**	-	**-**	-	-
Hemorrhage	-	-	-	-	11	**2.795**	-	**-**	-	**-**	-	-
Stroke	-	-	-	-	10	1.364	-	**-**	-	**-**	-	-
Abdominal Pain	-	-	-	-	-	**-**	10	**5.226**	**3**	**4.940**	-	-
Dysphagia	-	-	-	-	-	-	6	**7.113**	-	**-**	-	-
Diabetes Mellitus	-	-	-	-	-	-	4	**4.871**	-	**-**	-	-
Myasthenia	-	-	-	-	-	-	4	**4.076**	-	**-**	-	-
Cardiomyopathy	-	-	-	-	-	**-**	**4**	**78.025**	**2**	**73.329**	-	-
Cardiac Arrest	-	-	-	-	-	**-**	**3**	**3.824**	-	**-**	4	**5.509**
Constipation	-	-	-	-	-	**-**	**3**	1.693	-	**-**	-	-
Hypotension	-	-	-	-	-	**-**	**3**	1.688	-	**-**	-	-
Malaise	-	-	-	-	-	**-**	**3**	0.694	-	**-**	-	-
Pulmonary Embolism	-	-	-	-	-	**-**	**3**	**3.092**	-	**-**	-	-
Myelosuppression	-	-	-	-	-	**-**	**-**	**-**	12	**248.377**	38	**272.420**
Neurotoxicity	-	-	-	-	-	**-**	**-**	**-**	3	**67.316**	-	-
Abdominal Distension	-	-	-	-	-	**-**	**-**	**-**	2	**7.331**	3	**3.684**
Chest Pain	-	-	-	-	-	**-**	**-**	**-**	2	**7.508**	-	-
Chills	-	-	-	-	-	**-**	**-**	**-**	2	**6.428**	-	-
Cough	-	-	-	-	-	**-**	**-**	**-**	2	**2.751**	3	1.383
Gastrointestinal Dysfunction	-	-	-	-	-	**-**	**-**	**-**	2	**8.754**	3	**4.399**
Palmar-Plantar Erythrodysaesthesia Syndrome	-	-	-	-	-	**-**	**-**	**-**	-	**-**	8	**38.775**
Mouth Ulceration	-	-	-	-	-	**-**	**-**	**-**	-	**-**	6	**35.835**
Hypokalemia	-	-	-	-	-	**-**	**-**	**-**	-	-	5	**14.982**
Leukopenia	-	-	-	-	-	**-**	**-**	**-**	-	-	5	**13.218**
Allergic Dermatitis	-	-	-	-	-	**-**	**-**	**-**	-	-	4	**41.507**
Peripheral Edema	-	-	-	-	-	**-**	**-**	**-**	-	-	4	**3.623**
Drug-Induced Liver Injury	-	-	-	-	-	**-**	**-**	**-**	-	-	3	**12.436**
Granulocytopenia	-	-	-	-	-	**-**	**-**	**-**	-	-	3	**59.203**
Total	21,276		22,415		78		138		114		203	

ROR, reported odds ratio. Bolded text indicates statistical significance (ROR > 2, which means that the odds for this adverse event when using the drug is at least twice as likely as for all other drugs) PD-L1.

**Table 2:** **PD-L1 Immune Checkpoint Inhibitor Adverse Events.** Reported odds ratio (ROR) for the 25 most common adverse events associated with each anti-PD-L1 immune checkpoint inhibitor.

Adverse Event	Atezolizumab		Avelumab		Durvalumab	
	Events	ROR	Events	ROR	Events	ROR
Pyrexia	630	**3.475**	47	**2.422**	136	**2.500**
Diarrhoea	530	1.481	72	1.932	76	0.704
Fatigue	461	1.016	62	1.31	72	0.528
Anaemia	455	**4.549**	-	-	65	**2.153**
Interstitial Lung Disease	433	**17.187**	43	**15.905**	151	**20.135**
Hypothyroidism	367	**23.193**	51	**30.184**	68	**14.087**
Nausea	352	0.727	49	0.969	48	0.331
Pneumonia	343	**2.031**	24	1.337	86	1.714
Rash	339	1.359	41	1.566	74	0.997
Neutropenia	939	**7.213**	-	-	339	**5.916**
Decreased appetite	329	2.709	28	**2.175**	51	1.402
Dyspnoea	310	0.948	30	0.869	117	1.217
Thrombocytopenia	516	**4.269**	26	**4.052**	113	**3.972**
Pneumonitis	294	**21.799**	22	**15.05**	215	**55.300**
Hypertension	293	**2.418**	47	**3.719**	-	-
Asthenia	263	1.245	24	1.076	43	0.682
Hepatic Dysfunction	255	**12.471**	48	**12.128**	56	**9.120**
Proteinuria	241	**23.134**	-	-	**-**	**-**
Ascites	240	**15.186**	-	-	**-**	**-**
Vomiting	238	0.859	30	1.032	-	-
Adrenal insufficiency	231	**42.411**	35	**58.993**	**-**	**-**
Acute kidney injury	224	**2.484**	29	**3.061**	**-**	**-**
Colitis	220	**11.824**	38	**19.384**	72	**12.996**
Sepsis	200	**3.321**	-	-	61	**3.420**
Constipation	195	1.687	-	-	-	-
Chills	-	-	35	**5.151**	-	-
Malaise	-	-	35	1.19	-	-
Peripheral Neuropathy	-	-	35	**6.359**	-	**-**
Myocarditis	-	-	27	**43.085**	58	**33.018**
Pruritus	-	-	25	1.217	-	-
Renal Dysfunction	-	-	24	**4.446**	-	-
Myelosuppression	-	-	-	-	84	**24.192**
Pleural Effusion	-	-	-	-	59	**5.971**
Pancytopenia	-	-	-	-	39	**4.386**
Abdominal Pain	-	-	-	-	38	0.99
Pruritus	-	-	-	-	38	0.654
Myositis	-	-	-	-	36	**24.739**
Total	8898		927		2195	

ROR, reported odds ratio. Bolded text indicates statistical significance (ROR > 2), which means that the odds for this adverse event when using the drug are at least twice as likely as for all other drugs).

**Table 3:** **CTLA-4 and LAG-3 Immune Checkpoint Inhibitor Adverse Events.** Reported odds ratio (ROR) for the 25 most common adverse events associated with the anti-CTLA-4 and anti-LAG-3 immune checkpoint inhibitors.

Adverse Event	Ipilimumab (CTLA-4)		Tremelimumab (CTLA-4)		Relatlimab (LAG-3)	
	Events	ROR	Events	ROR	Events	ROR
Diarrhoea	1403	**2.870**	8	0.825	1	1.472
Colitis	1556	**61.885**	25	49.337	1	**183.992**
Pyrexia	966	**3.780**	42	9.327	2	**5.986**
Rash	944	**2.742**	-	-	-	-
Fatigue	635	0.987	7	0.571	1	1.168
Hypophysitis	893	**565.424**	-	-	-	-
Nausea	553	0.808	-	-	-	-
Adrenal Insufficiency	494	**68.453**	-	-	-	-
Decreased Appetite	493	**2.871**	-	-	-	-
Pruritus	419	1.523	-	-	-	-
Hepatic Dysfunction	1059	**12.086**	30	**72.700**	1	**35.265**
Hypothyroidism	412	**18.322**	-	-	-	-
Vomiting	411	1.051	9	1.230	-	-
Pneumonitis	400	**21.095**	13	35.865	-	-
Malaise	379	0.953	-	-	-	-
Pneumonia	360	1.494	-	-	5	**18.674**
Dyspnoea	352	0.755	18	2.122	-	-
Headache	332	0.602	-	-	-	-
Interstitial Lung Disease	295	**8.073**	-	-	-	-
Anaemia	293	**2.025**	-	-	-	-
Acute Kidney Injury	281	**2.195**	-	-	-	-
Dehydration	281	**2.835**	14	7.675	-	-
Asthenia	266	0.883	-	-	-	-
Sepsis	259	**3.033**	36	24.407	3	**29.050**
Arthralgia	253	0.777	-	-	1	**2.354**
Biliary Tract Infection	-	-	28	4275.104	-	-
Abdominal Pain	-	-	26	8.007	-	-
Myocarditis	-	-	15	96.574	3	**292.250**
Gastrointestinal Hemorrhage	-	-	12	7.875	-	-
Intestinal Perforation	-	-	12	70.125	-	-
Myasthenia	-	-	12	7.131	-	-
Pancytopenia	-	-	12	15.321	-	-
Drug-Induced Liver Injury	-	-	11	24.795	-	-
Confusion	-	-	11	3.866	-	-
Neutropenia	-	-	10	11.881	-	-
Hypotension	-	-	10	3.279	-	-
Presyncope	-	-	10	28.669	-	-
Nephritis	-	-	8	170.176	-	-
Chills	-	-	7	4.052	-	-
Myositis	-	-	7	53.599	2	**227.604**
Anxiety	-	-	-	-	4	**10.301**
Infection	-	-	-	-	4	**75.279**
Hyponatremia	-	-	-	-	2	**36.026**
Myasthenia Gravis	-	-	-	-	2	**344.496**
Pulmonary Edema	-	-	-	-	1	**190.840**
Arthritis	-	-	-	-	1	**12.184**
Third Degree Heart Block	-	-	-	-	1	**133.639**
Cardiogenic Shock	-	-	-	-	1	**65.547**
Confusion	-	-	-	-	1	**5.486**
Dermatitis	-	-	-	-	1	**55.556**
Dysphagia	-	-	-	-	1	**9.666**
ECG Abnormalities	-	-	-	-	1	**101.703**
Epilepsy	-	-	-	-	1	**29.521**
Hemophagocytic Lymphohistiocytosis	-	-	-	-	1	**178.250**
Hydrocephalus	-	-	-	-	1	**157.363**
Total	13,989		393		43	

ROR, reported odds ratio. Bolded text indicates statistical significance (ROR > 2, which means that the odds for this adverse event when using the drug is at least twice as likely as for all other drugs).

## Data Availability

All data utilized in the study is publicly available at https://openvigil.sourceforge.net (accessed on 2 June 2024) through the FDA Adverse Events Reporting System for free, and thus, no data is shared from this study.
